# Supplementary material for: Climate-driven deoxygenation elevates fishing vulnerability for the ocean's widest ranging shark
Source: eLife. 2021 Jan 19;10:e62508. doi: 10.7554/eLife.62508 (PMC7815312; doi:10.7554/eLife.62508)
Supplement: Supplementary file 1. — F, female; M, male. Shaded rows indicate colour-coded shark numbers in Figures 1 and 2; DNR, did not report. [file elife-62508-supp1.docx]

**Supplementary file 1. Summary data for satellite-tagged blue sharks**. F, female; M, male. Shaded rows indicate colour-coded shark numbers in Figures 1 and 2; DNR – did not report.

| **Shark ID** | **Fork length (cm)** | **Sex** | **Tagging location** | **Tagging date** | **PSAT Pop-up date** | **Days-at-liberty** | **Days at OMZ** | **Time at OMZ (%)** | **Tag type** |
| --- | --- | --- | --- | --- | --- | --- | --- | --- | --- |
| S1 | 175 | F | Azores | 26 Feb. 2009 | 13 Nov 2009 | 226 | 92 | 40.7 | ARGOS |
|  |  |  |  |  |  | 91 | 19 | 20.9 | PSAT |
| S2 | 127 | F | Azores | 19 Feb. 2009 |  | 879 | 14 | 1.6 | ARGOS |
| S3 | 139 | F | Azores | 10 Feb. 2009 |  | 618 | 0 | 0.0 | ARGOS |
| S4 | 142 | F | Azores | 26 Feb. 2009 |  | 28 | 0 | 0.0 | ARGOS |
| S5 | 145 | F | Azores | 26 Feb. 2009 |  | 954 | 0 | 0.0 | ARGOS |
| S6 | 148 | F | Azores | 26 Feb. 2009 |  | 92 | 0 | 0.0 | ARGOS |
| S7 | 168 | M | Azores | 16 Oct. 2009 |  | 37 | 0 | 0.0 | ARGOS |
| S8 | 133 | M | Azores | 16 Oct. 2009 |  | 207 | 0 | 0.0 | ARGOS |
| S9 | 201 | M | Azores | 16 Oct. 2009 |  | 163 | 0 | 0.0 | ARGOS |
| S10 | 156 | M | Azores | 16 Oct. 2009 |  | 83 | 0 | 0.0 | ARGOS |
| S11 | 130 | M | Azores | 02 Dec. 2009 |  | 44 | 0 | 0.0 | ARGOS |
| S12 | 178 | F | Azores | 02 Dec. 2009 |  | 581 | 0 | 0.0 | ARGOS |
| S13 | 164 | M | Azores | 20 Aug. 2010 |  | 246 | 114 | 46.3 | ARGOS |
| S14 | 140 | M | Azores | 20 Aug. 2010 |  | 127 | 0 | 0.0 | ARGOS |
| S15 | 183 | M | Azores | 20 Aug. 2010 |  | 526 | 0 | 0.0 | ARGOS |
| S16 | 211 | M | Azores | 20 Aug. 2010 |  | 528 | 0 | 0.0 | ARGOS |
| S17 | 159 | M | Azores | 20 Aug. 2010 |  | 214 | 34 | 15.9 | ARGOS |
| S18 | 172 | M | Azores | 20 Aug. 2010 |  | 370 | 0 | 0.0 | ARGOS |
| S19 | 180 | M | Azores | 20 Aug. 2010 |  | 382 | 96 | 25.1 | ARGOS |
| S20 | 207 | M | Azores | 20 Aug. 2010 |  | 200 | 0 | 0.0 | ARGOS |
| S21 | 183 | M | Azores | 20 Aug. 2010 |  | 117 | 44 | 37.6 | ARGOS |
| S22 | 201 | F | Azores | 18 May 2012 |  | 34 | 0 | 0.0 | ARGOS |
| S23 | 220 | F | Cape Verde | 16 Jan 2017 |  | 73 | 62 | 84.9 | ARGOS |
| S24 | 230 | M | Cape Verde | 17 Jan 2017 |  | 40 | 40 | 100.0 | ARGOS |
| S25 | 195 | F | Cape Verde | 17 Jan 2017 |  | 23 | 23 | 100.0 | ARGOS |
| S26 | 270 | M | Cape Verde | 19 Jan 2017 |  | 84 | 25 | 29.8 | ARGOS |
| **Shark ID** | **Fork length (cm)** | **Sex** | **Tagging location** | **Tagging date** | **PSAT Pop-up date** | **Days-at-liberty** | **Days at OMZ** | **Time at OMZ (%)** | **Tag type** |
| S27 | 225 | F | Cape Verde | 19 Jan 2017 |  | 25 | 25 | 100.0 | ARGOS |
| S28 | 230 | F | Cape Verde | 20 Jan 2017 |  | 109 | 24 | 22.0 | ARGOS |
| S29 | 210 | F | Cape Verde | 22 Jan 2017 |  | 125 | 22 | 17.6 | ARGOS |
| S30 | 220 | M | Cape Verde | 26 Jan 2017 |  | 89 | 53 | 59.6 | ARGOS |
| S31 | 230 | F | Cape Verde | 25 Jan 2017 |  | 103 | 8 | 7.8 | ARGOS |
| S32 | 225 | M | Cape Verde | 26 Jan 2017 |  | 172 | 18 | 10.5 | ARGOS |
| S33 | 260 | M | Oceanic | 21 Aug. 2011 | 20 Nov. 2011 | 91 | 0 | 0.0 | PSAT |
| S34 | 250 | M | Oceanic | 22 Aug. 2011 | 21 Dec. 2011 | 121 | 0 | 0.0 | PSAT |
| S35 | 240 | M | Oceanic | 26 Aug. 2011 | 13 Nov. 2011 | 79 | 0 | 0.0 | PSAT |
| S36 | 200 | M | Oceanic | 26 Aug. 2011 | 25 Dec. 2011 | 121 | 0 | 0.0 | PSAT |
| S37 | 185 | M | Oceanic | 26 Jun. 2010 | 24 Sep. 2010 | 90 | 0 | 0.0 | PSAT |
| S38 | 192 | F | Oceanic | 27 Jun. 2010 | 23 Aug. 2010 | 57 | 0 | 0.0 | PSAT |
| S39 | 240 | F | Oceanic | 21 Aug. 2011 | 20 Nov. 2011 | 91 |  |  | PSAT |
| S40 | 240 | F | Oceanic | 26 Aug. 2011 | 25 Dec. 2011 | 121 | 0 | 0.0 | PSAT |
| S41 | 260 | F | Oceanic | 29 Jun. 2010 | 28 Oct. 2010 | 121 | 0 | 0.0 | PSAT |
| S42 | 240 | F | Oceanic | 30 Jun. 2010 | 28 Jul. 2010 | 28 | 0 | 0.0 | PSAT |
| S43 | 200 | M | Oceanic | 25 Jun. 2010 | 10 Jul. 2010 | 15 | 0 | 0.0 | PSAT |
| S44 | 210 | M | Oceanic | 25 Jun. 2010 | 03 Jul. 2010 | 8 | 0 | 0.0 | PSAT |
| S45 | 235 | M | Oceanic | 25 Jun. 2010 | 23 Dec. 2010 | 181 | 0 | 0.0 | PSAT |
| S46 | 220 | F | Oceanic | 27 Aug. 2011 | 26 Dec. 2011 | 121 | 0 | 0.0 | PSAT |
| S47 | 220 | F | Oceanic | 28 Aug. 2011 | 01 Dec. 2011 | 95 | 20 | 21.1 | PSAT |
| S48 | 240 | F | Cape Verde | 21 Jan. 2017 | 23 Mar. 2017 | 61 | 15 | 24.6 | PSAT |
| S49 | 265 | M | Cape Verde | 17 Feb. 2017 | 07 Apr. 2017 | 49 | 49 | 100.0 | PSAT |
| S50 | 255 | F | Cape Verde | 24 Jan. 2017 | 26 Mar. 2017 | 61 | 5 | 8.2 | PSAT |
| S51 | 200 | F | Cape Verde | 24 Jan. 2017 | 25 Apr. 2017 | 91 | 10 | 11.0 | PSAT |
| S52 | 230 | F | Cape Verde | 25 Jan. 2017 | 26 May 2017 | 121 | 30 | 24.8 | PSAT |
| S53 | 230 | M | Cape Verde | 26 Jan. 2017 | 29 Apr. 2017 | 93 | 88 | 94.6 | PSAT |
| S54 | 230 | M | Cape Verde | 28 Jan. 2017 | 29 May 2017 | 121 | 3 | 2.5 | PSAT |
| S55 | 220 | F | Cape Verde | 28 Jan. 2017 | DNR | - | - | - | PSAT |
