# Supplementary material for: Climate-driven deoxygenation elevates fishing vulnerability for the ocean's widest ranging shark
Source: eLife. 2021 Jan 19;10:e62508. doi: 10.7554/eLife.62508 (PMC7815312; doi:10.7554/eLife.62508)
Supplement: Supplementary file 2. — Real blue shark movements were compared to correlated random walk models for those sharks tagged in the Azores that encountered the ETA OMZ off western Africa. Proportion test described in Jaine et al., 2014; ns, non-significance. [file elife-62508-supp2.docx]

**Supplementary file 2. Testing habitat selection of blue sharks above the eastern tropical Atlantic (ETA) OMZ**. Real blue shark movements were compared to correlated random walk models for those sharks tagged in the Azores that encountered the ETA OMZ off western Africa. Proportion test described in Jaine et al. (2014); ns, denotes non-significance.

| **Shark ID** | **Days above OMZ** | **Time above OMZ (%)** | **% times that real sharks spent ≥ time in OMZ area compared to models** | ***p*-value** |
| --- | --- | --- | --- | --- |
| S1 | 92 | 40.7 | 99 | < 0.001 |
| S2 | 14 | 1.6 | 97 | < 0.001 |
| S13 | 114 | 46.3 | 100 | < 0.001 |
| S17 | 34 | 15.9 | 100 | < 0.001 |
| S19 | 96 | 25.1 | 6 | ns |
| S21 | 44 | 37.6 | 100 | < 0.001 |
| S23 | 62 | 84.9 | 71 | < 0.001 |
| S24 | 40 | 100.0 | 0 | ns |
| S25 | 23 | 100.0 | 0 | ns |
| S26 | 25 | 29.8 | 0 | ns |
| S27 | 25 | 100.0 | 56 | ns |
| S28 | 24 | 22.0 | 6 | ns |
| S29 | 22 | 17.6 | 89 | < 0.001 |
| S30 | 53 | 59.6 | 10 | ns |
| S31 | 8 | 7.8 | 0 | ns |
| S32 | 18 | 10.5 | 30 | ns |
