# Supplementary material for: Climate-driven deoxygenation elevates fishing vulnerability for the ocean's widest ranging shark
Source: eLife. 2021 Jan 19;10:e62508. doi: 10.7554/eLife.62508 (PMC7815312; doi:10.7554/eLife.62508)
Supplement: Supplementary file 3. — Shaded rows denote FAO area encompassing a permanent oxygen minimum zone. [file elife-62508-supp3.docx]

**Supplementary file 3. Fishing activity information for the different major fishing areas in the Atlantic**. Shaded rows denote FAO area encompassing a permanent oxygen minimum zone.

|  | **FAO fishing area** | **Mean number of fishing days (per grid cell)** | **Fishing intensity (%)** |
| --- | --- | --- | --- |
| N. Atlantic | FAO21 | 108.6 | 69.9 |
|  | FAO27 | 68.2 | 57.3 |
|  | FAO31 | 42.9 | 62.7 |
|  | FAO34 | 65.3 | 59.9 |
|  | FAO41 | 17.0 | 41.6 |
| S. Atlantic | FAO34 | 23.3 | 56.2 |
|  | FAO41 | 25.9 | 53.2 |
|  | FAO47 | 29.6 | 57.4 |
