# Supplementary material for: Climate-driven deoxygenation elevates fishing vulnerability for the ocean's widest ranging shark
Source: eLife. 2021 Jan 19;10:e62508. doi: 10.7554/eLife.62508 (PMC7815312; doi:10.7554/eLife.62508)
Supplement: Transparent reporting form [file elife-62508-transrepform.docx]

***eLife’s* transparent reporting form**

We encourage authors to provide detailed information *within their submission* to facilitate the interpretation and replication of experiments. Authors can upload supporting documentation to indicate the use of appropriate reporting guidelines for health-related research (see [EQUATOR Network](http://www.equator-network.org/%20)), life science research (see the [BioSharing Information Resource](https://biosharing.org/)), or the [ARRIVE guidelines](http://www.plosbiology.org/article/info:doi/10.1371/journal.pbio.1000412) for reporting work involving animal research. Where applicable, authors should refer to any relevant reporting standards documents in this form.

If you have any questions, please consult our Journal Policies and/or contact us: [editorial@elifesciences.org](mailto:editorial@elifesciences.org).

**Sample-size estimation**

- You should state whether an appropriate sample size was computed when the study was being designed
- You should state the statistical method of sample size computation and any required assumptions
- If no explicit power analysis was used, you should describe how you decided what sample (replicate) size (number) to use

Please outline where this information can be found within the submission (e.g., sections or figure legends), or explain why this information doesn’t apply to your submission:

Our submission reports individual movements and environmental data of wild pelagic sharks as determined with animal-attached electronic tags. The overall sample size of sharks was dependent upon the reporting rates of tags and data transmissions via satellites. However, we deployed electronic tags on 55 blue sharks in the eastern N Atlantic. A recent published study (A. Sequeira et al. 2019 Ecol. Applic.) indicates deployment of 40 tags on a species within a region is generally sufficient to identify key movement patterns of marine megafauna.

**Replicates**

- You should report how often each experiment was performed
- You should include a definition of biological versus technical replication
- The data obtained should be provided and sufficient information should be provided to indicate the number of independent biological and/or technical replicates
- If you encountered any outliers, you should describe how these were handled
- Criteria for exclusion/inclusion of data should be clearly stated
- High-throughput sequence data should be uploaded before submission, with a private link for reviewers provided (these are available from both GEO and ArrayExpress)

Please outline where this information can be found within the submission (e.g., sections or figure legends), or explain why this information doesn’t apply to your submission:

Correlated random walk simulations: number of walks used are given in Methods section.

Modelling shark behavior and environment: number of MDD depth determinations given in Figure 3, and how outliers were treated given in Methods.

Fishing vessel effort and catch data: number of vessels given in Methods and number of longline sets analysed given in Table 4.

**Statistical reporting**

- Statistical analysis methods should be described and justified
- Raw data should be presented in figures whenever informative to do so (typically when N per group is less than 10)
- For each experiment, you should identify the statistical tests used, exact values of N, definitions of center, methods of multiple test correction, and dispersion and precision measures (e.g., mean, median, SD, SEM, confidence intervals; and, for the major substantive results, a measure of effect size (e.g., Pearson's r, Cohen's d)
- Report exact p-values wherever possible alongside the summary statistics and 95% confidence intervals. These should be reported for all key questions and not only when the p-value is less than 0.05.

Please outline where this information can be found within the submission (e.g., sections or figure legends), or explain why this information doesn’t apply to your submission:

We provide full statistical reporting (N, test used, mean and SD or medians) within the main text, Methods and Tables.

GAMM: statistics reported in Table 2..

Fishing effort: test results given in Results.

Habitat selection: reported in Supplementary file 2.

Catch data: quadratic models and statistics given in legend of Figure 9.

(For large datasets, or papers with a very large number of statistical tests, you may upload a single table file with tests, Ns, etc., with reference to sections in the manuscript.)

**Group allocation**

- Indicate how samples were allocated into experimental groups (in the case of clinical studies, please specify allocation to treatment method); if randomization was used, please also state if restricted randomization was applied
- Indicate if masking was used during group allocation, data collection and/or data analysis

Please outline where this information can be found within the submission (e.g., sections or figure legends), or explain why this information doesn’t apply to your submission:

This does not apply to our submission since our data was from free-ranging sharks and fishing vessels.

**Additional data files (“source data”)**

- We encourage you to upload relevant additional data files, such as numerical data that are represented as a graph in a figure, or as a summary table
- Where provided, these should be in the most useful format, and they can be uploaded as “Source data” files linked to a main figure or table
- Include model definition files including the full list of parameters used
- Include code used for data analysis (e.g., R, MatLab)
- Avoid stating that data files are “available upon request”

Please indicate the figures or tables for which source data files have been provided:

All data needed to assess the conclusions are available in the main paper and Supporting Data. The data files are available to download from GitHub:

https://github.com/GlobalSharkMovement/BlueSharkOMZ/

The data files made available with the paper on GitHub are:

(1)	Raw summary shark dive data both inside and outside the OMZ area (shark_dive_data.csv);

(2)	Figure 1. Raster of DO at 100m used in shark movement/dive analysis at 0.25 × 0.25° (do_av2009-2016_100m.csv);

(3)	Figure 6D. Raster of the shark MDD depth for present-day at 1 × 1° for the area analysed (shark_MDD_prst_fao.csv);

(4)	Figure 7. Raster of vessel monitoring system (VMS) fishing effort data at 1 × 1° (VMS_f-effort.csv);

(5)	Raster of vessel monitoring system (VMS) fishing intensity data at 1 × 1° (VMS_f-intens.csv);

(6)	Figure 8A. Raster of Spanish longline logbook catch-per-unit-effort data at 1 × 1° for the area analysed (cpue_spanishLL.csv).
